# Combinatorial Genomic Biomarkers Associated with High Response in IgE-Dependent Degranulation in Human Mast Cells

**DOI:** 10.3390/cells13151237

**Published:** 2024-07-23

**Authors:** Issan Yee San Tam, Tak Hong Lee, Hang Yung Alaster Lau, See-Ying Tam

**Affiliations:** 1 School of Biomedical Sciences, Faculty of Medicine, The Chinese University of Hong Kong, Shatin, Hong Kong; iystam@hku.hk (I.Y.S.T.); hyalau@cuhk.edu.hk (H.Y.A.L.); 2 Allergy Centre, Hong Kong Sanatorium and Hospital, Happy Valley, Hong Kong; takhong.lee@hksh.com; 3 Department of Pathology, Stanford University School of Medicine, Stanford, CA 94305, USA

**Keywords:** inflammation, allergy, immunoglobulin E, mast cell degranulation, integrated network analysis, genomic biomarkers, precision medicine diagnostics

## Abstract

Mast cells are the major effector cells that mediate IgE-dependent allergic reactions. We sought to use integrated network analysis to identify genomic biomarkers associated with high response in IgE-mediated activation of primary human mast cells. Primary human mast cell cultures derived from 262 normal donors were categorized into High, Average and Low responder groups according to their activation response profiles. Transcriptome analysis was used to identify genes that were differentially expressed in different responder cultures in their baseline conditions, and the data were analyzed by constructing a personalized perturbed profile (PEEP). For upregulated genes, the construction of PEEP for each individual sample of all three responder groups revealed that High responders exhibited a higher percentage of “perturbed” samples whose PEEP values lay outside the normal range of expression. Moreover, the integration of PEEP of four selected upregulated genes into distinct sets of combinatorial profiles demonstrated that the specific pattern of upregulated expression of these four genes, in a tandem combination, was observed exclusively among the High responders. In conclusion, this combinatorial approach was useful in identifying a set of genomic biomarkers that are associated with high degranulation response in human mast cell cultures derived from the blood of a cohort of normal donors.

## 1. Introduction

Mast cells are the major effector cells of the immune system that mediate IgE-dependent allergic and hypersensitivity reactions, as well as innate and adaptive immunity [1,2,3,4,5,6]. High-affinity IgE receptors (FcεRI) are expressed on the surface of mast cells, and the crosslinking of FcεRI by allergen binding to the cell-bound allergen-specific IgE results in mast cell degranulation. Such FcεRI-dependent degranulation causes the immediate release of preformed granule mediators, such as histamine, followed by the synthesis and release of diverse lipid mediators and cytokines [5,7]. Mast cells reside in all tissues and are particularly abundant in barrier sites such as the skin, lung and gut; they are mostly derived from CD34+ pluripotent progenitor cells in the bone marrow, and these progenitors migrate into the blood and then reside in the tissues where they differentiate and mature under the influence of local environmental factors [8,9,10,11].

Stem cell factor (SCF), or c-kit ligand, was identified as the key mast cell growth factor and has been utilized to generate mature primary human mast cell cultures from pluripotent hematopoietic stem cells isolated from bone marrow, fetal liver, and cord blood and peripheral blood [12,13]. Specifically, many different protocols for generating human mast cells from the peripheral blood have been developed for human mast cell studies because of the readily accessibility to human donor peripheral blood. Moreover, these protocols have been widely used in many functional and mechanistic studies because the functional properties of such blood-derived human mast cells have been well characterized and validated [12,13,14,15,16,17,18,19,20,21,22,23,24,25,26,27,28,29,30]. This approach of using blood-derived human mast cells has been used in several recent studies that attempted to explore new technical investigative approaches for understanding human mast cell biology and to develop novel, innovative diagnostic tools for clinical allergy [27,30,31,32,33,34,35,36].

We have recently utilized peripheral-blood-derived mast cells generated from allergic patients for developing a patient-specific mast cell activation test (MAT) that was cross-referenced to a reference baseline database, which was originally constructed using a profile of IgE-dependent activation responses for mast cells derived from the peripheral blood of a cohort of healthy individual human donors [35]. Interestingly, the data collected for our reference baseline database revealed a high degree of functional heterogeneity in activation responses among mast cell cultures derived from our cohort. Specifically, we were able to categorize the full spectrum of activation responses into three different responder groups of High, Average and Low, which were defined by the values of mean ± standard deviation (SD) of the histamine release levels calculated for the activation responses [35]. 

Recent studies employing transcriptomic and epigenomic comparisons of peripheral blood-derived human mast cells with skin-derived and tonsillar mast cells have suggested that the peripheral blood-derived human mast cells recapitulate many unique functional genomic characteristics of human tissue-resident mast cells [32]. By taking advantage of these properties of the peripheral blood-derived human mast cells, we used the differential gene expression analysis to identify a set of “signature” response genes that were upregulated or downregulated in mast cells of the High responders as compared to those of the Low and Average responders. Furthermore, we employed the approach of personalized perturbation profile (PEEP) analysis, recently outlined by Menche et al. [37], to further analyze our differential gene expression data. The profiling allowed us to integrate the PEEP of a set of 4 selected genes into a distinct combinatorial gene expression pattern, or a barcode, for each individual sample. We showed that the specific pattern of upregulated expression of these 4 genes in a tandem combination was observed exclusively among the High responders. Our studies thus revealed that PEEP profiling can be used to identify genomic biomarkers that are associated with high degranulation response in mast cell cultures derived from a cohort of normal healthy human donors. 

## 2. Materials and Methods

### 2.1. Normal Human Buffy Coats and Allergic Patient Peripheral Blood Samples

The protocol of using human buffy coats in our studies was approved by The Chinese University of Hong Kong Joint CUHK-NTEC Clinical Research Ethics Review Committee (CREC #2016.421). Fresh human buffy coats from unidentified healthy adult donors were obtained from the Hong Kong Red Cross within 24 h after blood donation. The studies employing human peripheral blood derived from allergic patients (SH#1, SH#5, SH#7) recruited from Hong Kong Sanatorium and Hospital were conducted as described in the Hospital Ethics Committee approved clinical trial (REC-2017-17) and registered at ClinicalTrials.Gov (Identifier NCT03406325). Written informed consent was obtained from each patient participant as stated in the protocol. For patients SH#1, SH#5 and SH#7, about 70–100 mL of peripheral venous blood from individual patients were drawn by syringes and collected in a blood-collecting tube containing EDTA, which was promptly processed within 2 h for the generation of human cultured mast cells.

### 2.2. Generation of Primary Human-Cultured Mast Cells

Primary human cultured mast cells with characteristics of MC_TC_ phenotypes were generated using the protocol previously described by our group [25]. Briefly, CD34^+^ progenitors isolated from fresh adult buffy coat using MACS system (Miltenyi Biotec, Auburn, CA, USA) were cultured in complete IMDM (Iscove’s modified Dulbecco’s medium with 1X insulin–transferrin–selenium, 1X penicillin–streptomycin, 50 µM 2-mercaptoethanol and 0.1% BSA) with 200 ng/mL human SCF (PeproTech, Cranbury, NJ, USA), 100 ng/mL IL-6 and 1 ng/mL IL-3 for one week. Subsequently, the spent medium was replaced by complete IMDM with 200 ng/mL SCF, 100 ng/mL IL-6 and 15 ng/mL IL-9 for another week. For these initial 2 weeks of culturing, cells were incubated in hypoxic conditions (5% CO_2_, 5% O_2_, 37 °C). After the first 2 weeks, the spent medium was replaced by complete IMDM with 200 ng/mL SCF and 100 ng/mL IL-6 weekly for another two weeks. In week 5, the spent medium was replaced by complete IMDM with 200 ng/mL SCF, 100 ng/mL IL-6 and 10 ng/mL IL-4. From week 6 and thereafter, cells were cultured in complete IMDM with 100 ng/mL SCF and 50 ng/mL IL-6. From week 3 onwards, these cultures were incubated at normoxic conditions (5% CO_2_, 21% O_2_, 37 °C). After week 6, fully mature and homogenous populations of primary human MC_TC_ mast cells were generated and ready for functional studies.

### 2.3. Mast Cell Activation Assay

Release of histamine from mature human cultured mast cells (cultured for 9 weeks) was measured as an index of mast cell activation, as previously described [25]. Briefly, cells were harvested, seeded and sensitized with 0.5 µg/mL human myeloma IgE overnight in complete IMDM with 100 ng/mL SCF and 50 ng/mL IL-6. Cells were then washed once in PBS and resuspended in full HEPES buffer (FHB: 137 mM NaCl, 5.56 mM glucose, 12 mM HEPES, 2.7 mM KCl, 0.4 mM NaH_2_PO_4_, 1 mM CaCl_2_ at pH 7.4) with 0.03% HSA and then challenged by anti-human IgE (stock: 1 mg/mL; dilution: 1:1000) (Sigma-Aldrich, St. Louis, MO, USA) for 30 min at 37 °C. For studies using substance P (SP), compound 48/80, ionomycin, or A23187 as the stimulus, human mast cells were washed and resuspended in FHB with 0.03% HSA and then treated with each compound at the selected concentrations for 30 min. Samples of cell-free supernatant and corresponding cell pellet were then collected by centrifugation at 4 °C. Cells incubated in buffer alone served as the controls for spontaneous histamine release. Histamine contents in mast cell pellets and supernatants were measured spectrofluorometrically using Bran+Luebbe AutoAnalyzer 3 (Bran+Luebbe, Norderstedt, Germany). Histamine release was expressed as a percentage of the total cellular contents of histamine (sum of supernatant content and cell pellet content) that were released into the supernatant [histamine release (%)]. The results were corrected for the corresponding control spontaneous release of histamine in buffer alone.

### 2.4. Transcriptome Profiling

Assessment of levels of protein-coding and long intergenic non-coding RNA transcripts was performed using GeneChip Human Gene 2.0 ST Array (Affymetrix, Sunnyvale, CA, USA). Microarray data were analyzed using Partek Genomics Suite 6.6 software (Chesterfield, MO, USA). Affymetrix CEL files were loaded using the Robust Multi-Chip Average (RMA) method. Unsupervised sample clustering was performed using Principal Component Analysis (PCA). Group-wise differential gene expression analyses among High, Average and Low responders were performed using Mixed Model ANOVA using the Restricted Maximum Likelihood Estimation (REML) method. Lists of differentially expressed genes were created using the cutoff criteria of unadjusted *p*-value < 0.05 and fold-change > 1.2 or <−1.2 for a specific group comparison in the 5-way ANOVA.

### 2.5. Validation of Differential Gene Expression Data by Real-Time qPCR

Each individual cDNA sample derived from each donor culture of human mast cells was labeled as High, Average, or Low responder according to its activation profile. Transcripts of the target genes and reference gene (GAPDH) in each cDNA sample were quantified using TaqMan probes, TaqMan Fast Advanced Master Mix (Applied Biosystems, Foster City, CA), and Applied Biosystems QuantStudio 7 Flex Real-Time PCR System (Life Technologies, Carlsbad, CA, USA). QPCR Human Reference Total RNA (Cat.#750500, Agilent, Santa Clara, CA, USA) was used to prepare the standard curves. Quantities of the gene transcripts were converted from Ct values and calculated according to the standard curves using QuantStudio Software v1.3 (Life Technologies, Carlsbad, CA, USA). Expression level of a target gene in an individual cDNA sample was calculated as follows:

Expression level = (Qs/NFq)/(Gs/NFg)(1)

where

NFq = Qc/Average of all Qc;

NFg = Gc/Average of all Gc;

Qs = Mean quantity of the triplicate for target gene of each individual sample;

Qc = Mean quantity of the triplicate for target gene of plate-to-plate reference cDNA control;

NFq = Normalization factor for a plate (ratio of Qc/Average of all Qc for that target gene);

NFg = Normalization factor for a plate (ratio of Gc/Average of all Gc);

Gs = Mean quantity of the triplicate for GAPDH of each individual sample;

Gc = Mean quantity of the triplicate for GAPDH of plate-to-plate reference cDNA control.

The normalized expression level was then Log2 converted for each sample for each target gene. Using this method, an upregulated gene expression would give a positive Log2-converted expression level, whereas a down-regulated gene expression would give a negative value.

TaqMan probes used are listed as the following: AKAP12 (Hs00374507_m1), ARHGAP15 (Hs00251227_m1), CALB2 (Hs00242372_m1), CEBPD (Hs00270931_s1), DPP4 (Hs00897386_m1), FCGR1A/B/CP (Hs02340030_m1), FOXF1 (Hs00230962_m1), GADD45B (Hs00169587_m1), GAPDH (Hs03929097_g1), IL13AR1 (Hs00609812_m1), IL1R1 (Hs00991010_m1), IRF1 (Hs00971965_m1), ITM2C (Hs00985194_g1), KCNMA1 (Hs01119504_m1), NELL2 (Hs00196254-m1), PTGDR (Hs00235003_m1), SIGLEC8 (Hs00274289_m1), SOCS2 (Hs00919620_m1), SPTLC3 (Hs00217867_m1), THEMIS (Hs01041269_m1), TMEM255B (Hs00415678_m1).

### 2.6. Personalized Perturbation Profile (PEEP) Analysis

In our analysis, the absolute mRNA levels for individual samples were calculated using a human reference RNA standard and then normalized with the corresponding intrinsic GAPDH expression values. The absolute values of expression levels were then transformed by Log2 conversion, and the mean and standard deviation (SD) of the combined Average and Low (Ave + Low) responders for each gene were calculated. A “normal range” of expression levels was defined as those levels whose values lay between mean + SD and mean—SD. For a particular gene of an individual sample whose expression level value was found to be outside of the “normal range” of expression levels calculated for the combined (Ave + Low) responder groups, this gene is defined as being “perturbed”, as defined by the primary PEEP model outlined by Menche et al. [37]. Furthermore, the expression level of a gene was labeled as “Up” if it was higher than the upper limit of the “Normal range”, whereas those labeled “Down” had levels that were lower than the lower limit of the “Normal” range. The mean Log2-converted expression level and the SD for the 8 selected genes were calculated for High responders (*n* = 19) and the remaining donors (Average and Low responders, *n* = 32). The population-specific cutoff for perturbation defined for each gene was calculated as follows:

Up-regulation cutoff = M_Ave_Low_ + SD_Ave_Low_

Down-regulation cutoff = M_Ave_Low_ − SD_Ave_Low_

If M_D_ > Up-regulation cutoff, that donor would be classified as “Up” for that gene;

If M_D_ < Down-regulated cutoff, that donor would be classified as “Down” for that gene.

Total number of “Perturbed” observed = number of “Up” + number of “Down”;

where:

M_Ave_Low_ = Mean Log2-converted expression level for Average and Low responders;

SD_Ave_Low_ = Log2-converted expression level standard deviation for Average and Low responders;

M_D_ = Mean Log2-converted expression level of an individual donor.

## 3. Results

### 3.1. Construction of Reference Baseline Database of Activation Responses of Human-Cultured Mast Cells Derived from a Cohort of Normal Healthy Donors

**Figure 1:**
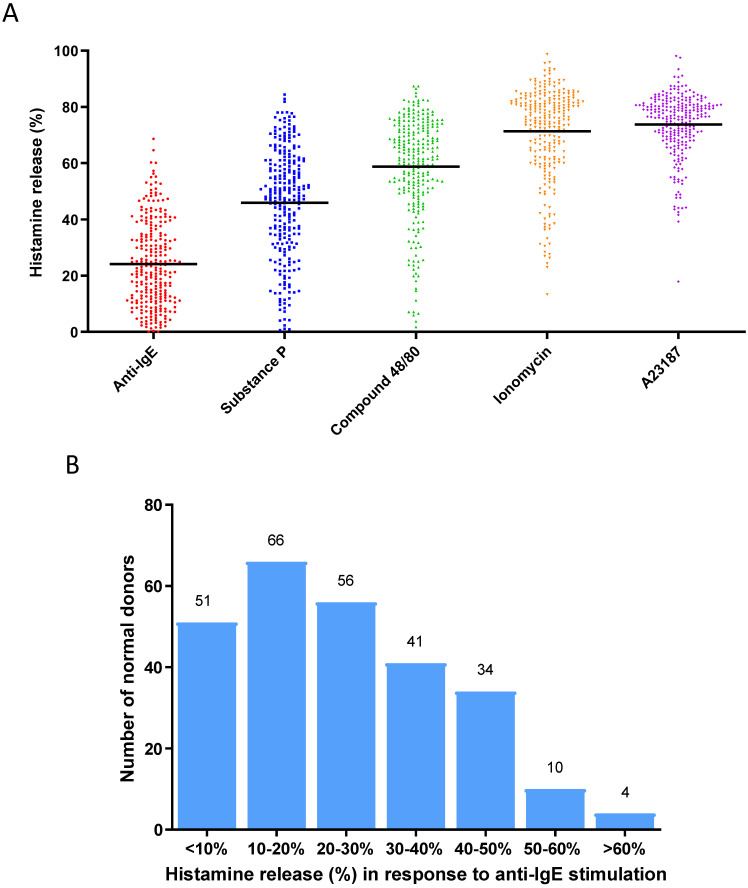
(**A**) Degranulation responses (histamine releases) induced by activation by different stimuli for 30 min in human mast cell cultures derived from a cohort of normal blood donors (*n* = 262): anti-IgE (1 µg/mL), substance P (10 µM), compound 48/80 (1 µg/mL), ionomycin (1 µM), A23187 (1 µM). Each dot of different colors shown in the figure represents a single normal individual donor. (**B**) Distribution of anti-IgE-induced degranulation responses among human mast cultures derived from a cohort of normal blood donors (*n* = 262). The numbers on the top of the boxes indicate the numbers of donors whose responses were within the range of % histamine release represented by each box.

To establish a population of primary human mast cell cultures derived from a cohort of normal healthy donors, we generated in vitro cultured mast cells from 262 normal donor buffy coats using a protocol previously reported by our group [25]. Mature mast cell cultures were subjected to functional characterization by activating them with different stimuli, such as anti-IgE (to induce the FcεRI-dependent cell activation) [5], SP (an agonist that induces MRGPRX2 receptor-dependent cell activation) [5], compound 48/80 (an agonist that induces MRGPRX2 receptor-dependent cell activation) [5], ionomycin (a calcium ionophore that induces calcium-dependent cell activation) [25], and A23187 (a calcium ionophore that induces calcium-dependent cell activation) [25], in order to present a broad functional characterization of these mast cell cultures by showing that these mast cell cultures were indeed fully functional mature and active in response to different selected stimuli (Figure 1A). When the anti-IgE-induced degranulation response was further analyzed for this cohort of donors, these mast cell cultures exhibited an unimodal distribution of histamine release response (Figure 1B): % histamine release [mean ± SD] was 24.1 ± 15.1%. We defined the range for Average responders to be between 9.0% and 39.2% (*n* = 161), which represents the two values that are ± one SD from the mean. Donors with responses < 9.0% release were classified as Low responders (*n* = 50), whereas those with responses > 39.2% were classified as High responders (*n* = 51) [35].

### 3.2. Gene Expression Analysis of Human Mast Cell Cultures Derived from Selected High, Average and Low Responders under Basal Unstimulated Conditions

**Figure 2:**
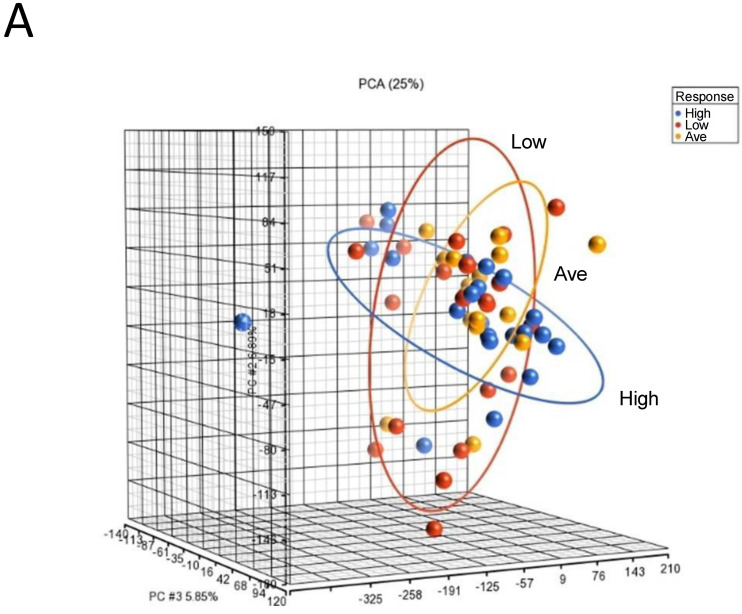

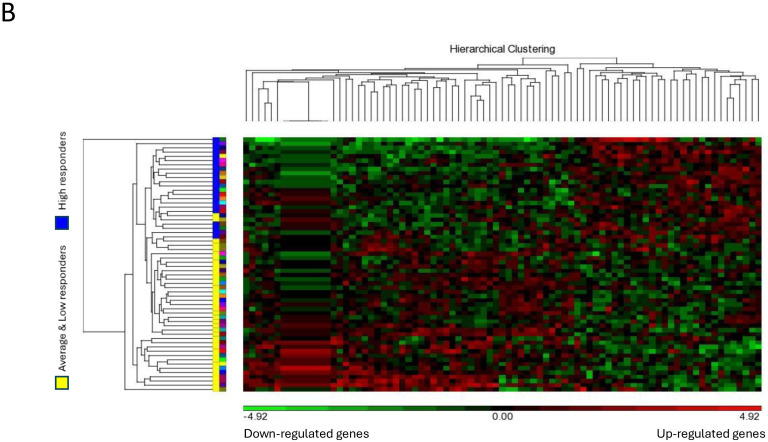

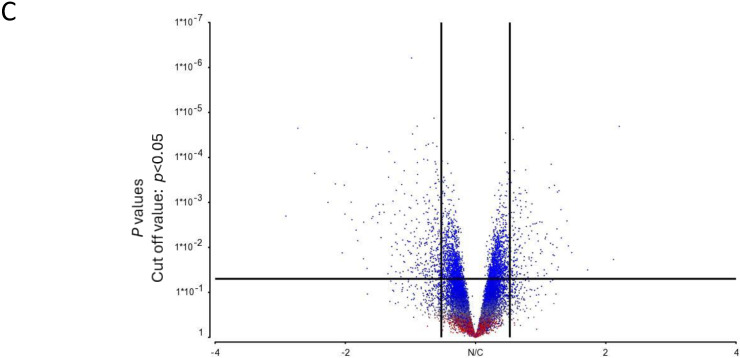
Microarray analysis of selected unstimulated mast cell cultures derived from High, Average and Low responder groups. (**A**) PCA mapping of genes expressed in all the selected samples showing the different gene expression patterns among unstimulated High, Average and Low responder mast cell cultures. (**B**) Heat-map analysis constructed by hierarchical clustering of the enriched gene list. Column: genes upregulated are shown in red, and genes downregulated are shown in green. Row: High responders are represented in blue; Average and Low responders are represented in yellow. (**C**) Volcano plot showing genes that were differentially expressed according to their respective responder groups. X-axis: fold-changes in gene expression by comparing High vs. Average and Low responders; cutoff values set at >1.2 or <–1.2. Y-axis: *p* values with a cutoff value set at *p* < 0.05.

To identify genomic biomarkers that were associated with high response in IgE-mediated activation in human mast cells, we compared the patterns of gene expression of human mast cell cultures under basal unstimulated conditions that were derived from selected donors of each responder group. We chose to assess the gene expression of the unstimulated cultures, as we reasoned that the gene expression profiles under the basal unstimulated conditions would directly reflect the different intrinsic transcriptional genomic programs that contributed to different genomic predispositions underlying the expression of differential functional responses in mast cell cultures derived from different responder groups. RNAs were isolated from selected unstimulated mast cell cultures derived from 22 High, 17 Average and 21 Low responders and then subjected to microarray gene expression analysis (total *n* = 60) (Figure S1). PCA mapping of genes expressed in the 60 selected samples showed that Average responders and Low responders shared a similar gene expression pattern than that of High responders, which was represented by the blue ellipse, whose plane was tilted at a different angle than those representing the Low and Average responder groups (Figure 2A).

The heat-map analysis, as illustrated in Figure 2B, showed that the patterns of gene expression of the enriched gene list were largely clustered into two main groups under the categories of “High responders” and “Average and Low responders”. These data were thus consistent with our PCA mapping results. In addition, genes differentially expressed in High responders vs. Average and Low responders were enlisted using a volcano plot. By selecting unadjusted *p* < 0.05 and fold-change > 1.2 or < −1.2 as the cutoff values, the numbers of genes enlisted in the volcano plot were narrowed down from 31,135 to 289 (Figure 2C).

### 3.3. Real-Time qPCR Validation of Selected Microarray Data and Identification of a Specific Gene Set Associated with Mast Cells Derived from High Responders

We initially selected 15 differentially expressed genes based on their statistical significance and physiological relevance, and TaqMan qPCR was performed using cDNA derived from 20 selected donor samples (7 High, 7 Average, 6 Low responders). As shown in Table S1, we found a highly consistent correlation in the patterns of gene expression of High responders vs. Low responders between those determined by microarray analysis and those calculated using qPCR analysis: i.e., genes that were upregulated in High responders exhibited a positive slope, whereas genes that were down-regulated in High responders showed a negative slope, suggesting that the qPCR analysis provided a consistent and accurate approach for further validation of our microarray data.

**Figure 3:**
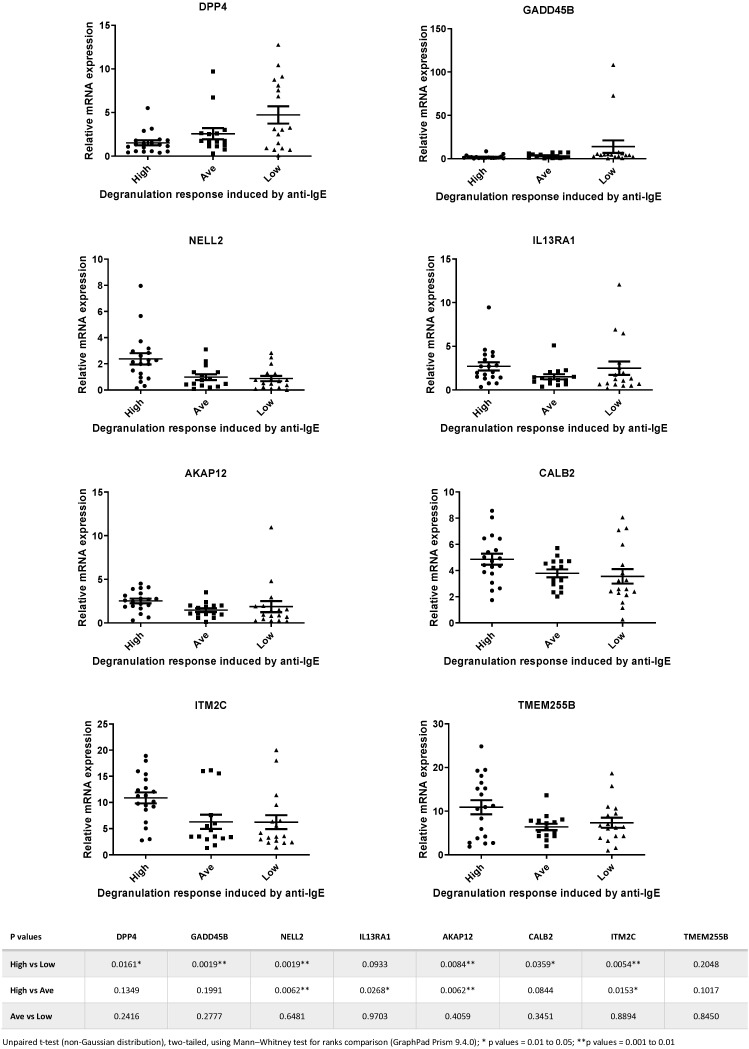
Relative expression levels of the 8 genes selected for TaqMan PCR analysis of samples from High, Average and Low responders. Statistical analysis revealed that significant differences in expression levels of DPP4, GADD45B, NELL2, IL13RA1, AKAP12, CALB2 and ITM2C were detected between High vs. Low responders and High vs. Average responders. Data calculated from the statistical analysis of the comparisons between different groups for each of the 8 genes are shown in the table beneath the figures.

Next, we selected 8 genes from the 15-gene list, and TaqMan qPCR analysis was performed with a larger number of samples (total *n* = 51). We picked these 8 genes based on the assumption that they are genes encoding signaling transduction factors and, therefore, are more likely to be involved in the IgE-receptor-mediated cell activation in human mast cells [5,33]. Figure 3 shows the expression levels of the 8 selected genes determined by TaqMan qPCR in samples that were derived from High, Average and Low responders. Statistical analysis revealed that significant differences in mRNA levels of DPP4, GADD45B, NELL2, IL13RA1, AKAP12, CALB2 and ITM2C were detected between High vs. Low responders and High vs. Average responders (Figure 3).

### 3.4. Analysis of Heterogeneity of Gene Expression Patterns by Constructing Personalized Perturbation Profiles

To characterize the heterogeneity of such gene expression patterns among different responders, we employed the method of personalized perturbation profile (PEEP) outlined by Menche et al. to construct the PEEP of a particular gene for each individual sample in order to characterize and compare the patterns of expression levels among different individuals in each responder group [37]. PEEP for each of the 8 selected genes was calculated for each individual sample in each responder group, and the “% perturbed”, “% up” and “% down” for each responder group were further determined (see “Materials and Methods” section for details) [37]. For genes such as AKAP12, CALB2, ITM2C, IL13RA1, NELL2 and TMEM255B, whose expression levels were upregulated in the High responders as compared to those in other groups (Figure 3), we found that their “% perturbed” and “% up” values in the High responders were higher than those calculated for other responder groups (Table 1). Similarly, for genes such as GADD45B and DPP4, whose expression levels were downregulated in the High responders as compared to those in other groups (Figure 3), we found that their “% perturbed” and “% up” values in the High responders were lower than those calculated for other responder groups (Table 1). These data thus suggest that the profiling using PEEP revealed the heterogeneity of expression levels of each of these genes in different responder groups and may provide a better assessment of how differential gene expression between the case and control groups can help to identify a set of genes as biomarkers.

### 3.5. Generation of Genomic Biomarkers Associated with High Mast Cell Activation Response Using a Combinatorial Model of a “Signature Gene Set” for Phenotypic Association of Human Mast Cell Cultures

We selected 4 genes from the 8-gene set and employed the PEEP data of these 4 genes as “modules” to construct a combinatorial model that would permit the association of phenotypic characteristics of human mast cells with high activation response. Specifically, we selected those 4 genes whose expressions were upregulated in High responders (AKAP12, ITM2C, IL13RA1, NELL2), and the PEEP of these 4 genes were integrated for every individual sample derived from the 3 different responder groups as “modules” to construct different combinatorial expression patterns. Such patterns were distinct for each individual sample using the perturbation criteria that were previously defined in our construction of PEEP: Expression of each gene in each individual sample was as follows: (1) within the normal range (Norm); (2) >upper limit of the normal range (Up); (3) <lower limit of the normal range (Down). Using such criteria, we would expect, in theory, 15 possible combinatorial patterns, or barcodes, each consisting of the 4 selected genes, that were represented in each individual sample (Table 2). These 15 different patterns were, in turn, classified into 6 “Categories” (Table 2).

From our normal donor cohort, we selected 3 individual mast cell samples (D#148, D#014, D#118) that belonged to each of the 3 responder groups, respectively, and assessed how the expression levels of these 4 genes in tandem combination in each sample would fall into each of the 6 “Categories”. As shown in Table 3, D#148 was a High responder to anti-IgE stimulation with upregulated expression of all 4 selected genes [4U0N0D], and thus the pattern fell into the category of “4-Up”. On the other hand, D#014 (Average responder) with the [0U2N2D] pattern and D#118 (Low responder) with the [0U4N0D] pattern both belonged to the category of “Others” (Table 3). In addition, we selected 3 mast cell samples (SH#1, SH#5, SH#7) from a group of allergic patients that were included in our recent study on mast cell activation test (MAT) using patient-derived mast cells [35]. We showed that [3U1N0D] (“3-Up”) was the pattern associated with the SH#7 mast cell culture, which had been determined to be a High responder. Furthermore, mast cell cultures derived from SH#1 and SH#5, which were both Average responders, exhibited the patterns of [0U4N0D] (“Others”) and [0U2N2D] (“Others”), respectively (Table 3). These data thus suggested that the combinatorial patterns of the expression levels of the 4 genes were highly correlated with the mast cell activation responses in both normal donors and allergic patients.

**Figure 4:**
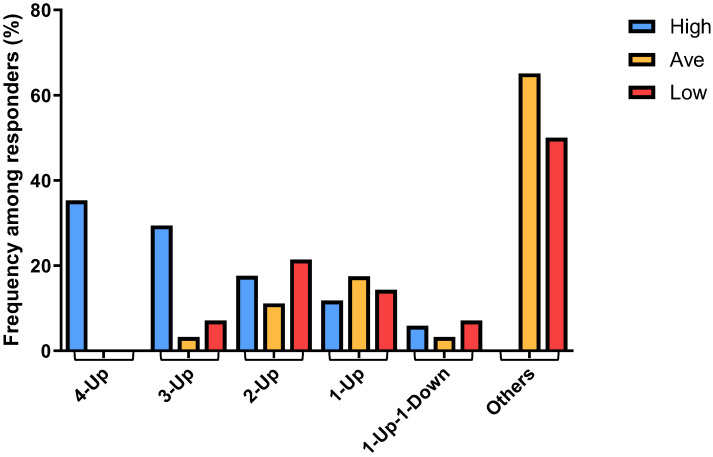
Different frequencies among different responders were determined for the combinatorial patterns comprising the 4 signature genes in each individual sample derived from High, Average and Low responder groups, showing the correlation of the patterns of differential numbers of upregulated genes with different mast cell activation responses among different responders.

By plotting the combinatorial patterns of the 4 signature genes in each individual sample derived from different responder groups versus the distribution frequencies of such patterns among each of the 3 responder groups, we found that the “4-Up” combinatorial pattern was associated exclusively with ~35% (6 out of 17) of the High responder group (Figure 4). Furthermore, the majority of the “3-Up” samples were found in ~30% (5 out of 17) of the High responders, whereas “2-Up” samples were found equally in all responder groups (Figure 4). On the other hand, the numbers of “1-Up” pattern samples were slightly higher in both Average and Low responders. It was of interest to note that we found only a few “1-Up-1-Down” samples, and all the other combinations (“Others”), such as those within the normal range and “4-Down”, were derived predominantly from ~68% (43 out of 63) of the Average and ~57% (8 out of 14) of the Low responders (Figure 4). These data thus suggested that the distribution of the patterns of combinatorial profiles of upregulated genes in different responder groups can be used as genomic biomarkers that are associated with high mast cell activation responses exhibited by the High responders.

## 4. Discussion

In the present study, we established a cohort of functionally characterized human mast cell cultures derived from the peripheral blood of 262 normal human donors for our transcription profiling studies. We further classified these cultures into High, Average and Low responder groups according to their anti-IgE-induced degranulation responses, and selected cultures were subjected to differential gene expression analysis. The rationale of our approach of employing a healthy human cohort dataset was consistent with that of a recent study by Bonaguro et al. [38], which used the variance of gene expression in healthy human cohorts to predict the role of individual genes or groups of genes in selected biological functions by integrating the expression, phenotypic and functional data from the cohort datasets. Using an approach designated as “*huva*” (“human variation”), this published study derived 2 experimental groups with High and Low expression of the gene of interest from a normal reference dataset, which allowed the subsequent comparisons of their transcriptional profiles and functional parameters, leading to the identification of phenotypic relevance of 16,000 genes-of-interest and stratification of genes according to their functions [38,39]. Thus, both their study and ours have demonstrated the wide applicability of using reference datasets of normal individuals for the elucidation of gene functions/phenotypes in functional genomics studies.

Many integrated network analysis tools have been developed and employed to analyze the transcriptomic data that are obtained using microarray or RNAseq techniques as the approaches to identify predictive biomarkers for patient stratification [40]. These approaches include unsupervised classification or supervised machine learning (such as Bayesian classifiers and support vector machine), differential expression gene analysis, differential network analysis, personalized perturbation profile (PEEP) analysis, and sample-specific network [40]. In this study, we identified large numbers of genes that were differentially regulated in the High responder mast cells compared to Average and Low responders, and 8 of these genes were selected for further analysis by constructing PEEP for each individual sample in each responder group. PEEP analysis is a bioinformatics tool recently introduced by Menche et al. both to address the limitations posed by the machine learning approaches and to systematically analyze the heterogeneity that is inherent in gene expression data [37]. In this approach, instead of comparing the mean of the case group vs. that of the control group, each case sample value is individually assessed on whether it lies within or outside (“perturbed”) the range of values calculated for the control samples [37]. For genes such as AKAP12, CALB2, ITM2C, IL13RA1, NELL2 and TMEM255B, whose expression levels were upregulated in the High responders as compared to those in other groups, we found that their “% perturbed” in the High responders were significantly higher than those calculated for other responder groups. On the other hand, for genes GADD45B and DPP4, whose expression levels were downregulated in the High responders as compared to those in other groups, we found that their “% perturbed” values in the High responders were significantly lower than those calculated for other responder groups. Consistent with the study by Menche et al., by comparing the PEEP of each test sample against the range of values derived from the controls, the heterogeneity inherent in such data can be better revealed than the conventional method of comparing the mean of the test samples against the mean of the control samples [37]. Moreover, importantly, the profiling using PEEP allowed us to further integrate the PEEP of a set of signature genes to form a combinatorial gene expression pattern or a barcode, for each individual sample, leading to the identification of a set of genomic biomarkers that was associated with specific functional characteristics of human mast cell cultures derived from a normal population of healthy donors.

In this study, we selected 4 upregulated genes from the 8-gene list and integrated the PEEPs of these 4 genes of each individual sample of all responders into different combinatorial patterns, or barcodes, which indicated the actual numbers of upregulated genes in each sample. As shown in Table 2, there are 15 possible combinatorial expression patterns (permutations) of each individual sample derived by combining the observed expression levels of each of the 4 genes. If we had chosen to perform PEEP profiling with 8 genes, the number of possible permutations of each individual sample derived by combining the observed expression levels of each of the 8 genes would be 45. In that case, the resulting data would be far too extensive and complicated to be analyzed for a proof-of-principle study such as ours. Indeed, in this study, we assessed the distribution of such combinatorial patterns among each of the 3 responder groups and found that the specific combinatorial pattern consisted of all 4 upregulated genes (“4-Up”) was found exclusively in ~35% of the High responder group (Figure 4). These data suggest that we can use the “4-Up” combinatorial pattern of the 4 selected genes as the specific genomic biomarker for the High responders of the IgE-mediated human mast cell activation, whereas the “Others” combinatorial patterns (see Table 2 for classification) can be used as the specific genomic biomarkers for the Low and Average responders of the IgE-mediated human mast cell activation. Our data are thus in line with the approach outlined by Menche et al. that the analysis using a distinct PEEP combinatorial perturbed gene expression pattern for each individual sample can be used to identify biomarkers that are associated with the functional status of that individual sample [37].

It will be of interest to determine further the regulatory mechanisms by which the coordinated upregulated expression of the 4 selected genes can confer the high activation response observed with the High responders. Indeed, both NELL2 (neural EGFL like 2) protein and AKAP12 (A-kinase anchoring protein 12) protein are known protein kinase C interacting partners [41,42], and protein kinase C has been shown to mediate IgE-dependent activation of mast cells [43,44]. Furthermore, it has been reported that IL-13 stimulation of human lung mast cells via IL13RA1 (interleukin 13 receptor subunit alpha 1) increases mast cell proliferation and FcεRI expression [45]. However, the role of ITM2C (integral membrane protein 2C) in mast cell activation has not been elucidated so far. In this context, reconstruction of gene regulatory networks (GRNs) has been proposed to address the relationship between the genes and their regulators, which is based on the rationale that every single gene may have a slight individual effect, but several genes disrupted together may have a strong, robust effect when they converge into some connected key regulatory pathways [46].

We have recently shown that peripheral-blood-derived mast cell cultures generated from different allergic patients exhibited extensive heterogeneity with differential activation/interaction of the high-affinity IgE receptors [35]. In that study, among the 7 allergic patients that were studied, we found that 5 of the mast cell cultures derived from these patients exhibited anti-IgE induced cell activation in the category of High responders by using the same criteria and the normal donor cohort that were employed in this present study [35]. Moreover, one of these High-responder patient mast cell cultures exhibited hyper-activation (>100th percentile of the cohort population) [35]. These findings thus suggest that high response in IgE-dependent mast cell activation may represent a genomic characteristic trait or signature response of mast cell cultures derived from allergic patients. Further implication can be extended to the apparently normal individuals in the general population who may be susceptible to mast cell-mediated allergic diseases because of their high mast cell activation profiles.

In the present study, as a preliminary attempt to validate our findings obtained with the PEEP analysis using our normal human cohort, we assessed whether our PEEP analysis could also be associated with the functional status of individual samples derived from an entirely different patient cohort. Indeed, with a limited sample size of 3 allergic patients that were derived from our previous allergic patient cohort [35], we were able to show that the combinatorial pattern of the expression levels of the 4 signature genes was closely correlated with the mast cell activation response observed in each of the 3 patient-derived cultures (Table 3). Further studies will be useful in identifying and developing specific genomic biomarkers that are specific for mast cells derived from different types of allergic patients as a new approach for patient stratification in the practice of personalized medicine.

## 5. Conclusions

A reference baseline dataset of IgE-dependent activation response of human mast cell cultures derived from a cohort of normal healthy donors was created, and the cultures were classified into 3 groups of responders: High, Average and Low. We constructed PEEP of a set of 4 genes that were differentially expressed at higher levels in the High responders and integrated such PEEP into combinatorial gene signatures that are associated with the high degranulation response in human mast cells. Our study thus represents a proof-of-principle to illustrate the concept of using both the reference baseline database and PEEP profiling as a novel approach for differential gene expression analysis and for the identification of genomic biomarkers associated with specific functional characteristics.

## 6. Patents

All the data presented in this report were included in a patent application filed with the U.S. Patent Office.

## Figures and Tables

**Table 1:** PEEP profiling revealed the heterogeneity of expression levels of each of the 8 selected genes in different responder groups. For AKAP12, CALB2, ITM2C, IL13RA1, NELL2 and TMEM255B genes, whose expression levels were upregulated in the High responders as compared to those in other groups, their “% perturbed” and “% up” values in the High responders were higher than those calculated for other responder groups. For GADD45B and DPP4 genes, whose expression levels were downregulated in the High responders as compared to those in other groups, their “% perturbed” and “% up” values in the High responders were lower than those calculated for other responder groups.

Gene Name	High	Ave	Low	Ave+Low	Total	Gene Name	High	Ave	Low	Ave+Low	Total
NELL2						CALB2					
Perturbed						Perturbed					
(N)	14	14	4	18	32	(N)	10	9	11	20	30
(%)	82.4%	22.2%	28.6%	23.4%	34.0%	(%)	58.8%	14.3%	78.6%	26.0%	31.9%
Up						Up					
(N)	14	8	2	10	24	(N)	10	7	3	10	20
(%)	82.4%	12.7%	14.3%	13.0%	25.5%	(%)	58.8%	11.1%	21.4%	13.0%	21.3%
Down						Down					
(N)	0	6	2	8	8	(N)	0	2	8	10	10
(%)	0.0%	9.5%	14.3%	10.4%	8.5%	(%)	0.0%	3.2%	57.1%	13.0%	10.6%
AKAP17						GADD45B					
Perturbed						Perturbed					
(N)	13	18	6	24	37	(N)	0	15	4	19	19
(%)	76.5%	28.6%	42.9%	31.2%	39.4%	(%)	0.0%	23.8%	28.6%	24.7%	20.2%
Up						Up					
(N)	12	9	3	12	24	(N)	0	5	3	8	8
(%)	70.6%	14.3%	21.4%	15.6%	25.5%	(%)	0.0%	7.9%	21.4%	10.4%	8.5%
Down						Down					
(N)	1	9	3	12	13	(N)	0	10	1	11	11
(%)	5.9%	14.3%	21.4%	15.6%	13.8%	(%)	0.0%	15.9%	7.1%	14.3%	11.7%
ITM2C						TMEM255B					
Perturbed						Perturbed					
(N)	13	22	3	25	38	(N)	10	12	6	18	28
(%)	76.5%	34.9%	21.4%	32.5%	40.4%	(%)	58.8%	19.0%	42.9%	23.4%	29.8%
Up						Up					
(N)	13	10	3	13	26	(N)	10	3	5	8	18
(%)	76.5%	15.9%	21.4%	16.9%	27.7%	(%)	58.8%	4.8%	35.7%	10.4%	19.1%
Down						Down					
(N)	0	12	0	12	12	(N)	0	9	1	10	10
(%)	0.0%	19.0%	0.0%	15.6%	12.8%	(%)	0.0%	14.3%	7.1%	13.0%	10.6%
IL13RA1						DPP4					
Perturbed						Perturbed					
(N)	10	14	4	18	28	(N)	4	18	8	26	30
(%)	58.8%	22.2%	28.6%	23.4%	29.8%	(%)	23.5%	28.6%	57.1%	33.8%	31.9%
Up						Up					
(N)	10	6	3	9	19	(N)	2	6	8	14	16
(%)	58.8%	9.5%	21.4%	11.7%	20.2%	(%)	11.8%	9.5%	57.1%	18.2%	17.0%
Down						Down					
(N)	0	8	1	9	9	(N)	2	12	0	12	14
(%)	0.0%	12.7%	7.1%	11.7%	9.6%	(%)	11.8%	19.0%	0.0%	15.6%	14.9%
Sample Profiles											
				**Mean**				**Standard Deviation**			
Histamine release (%)				26.96%				0.1530			
Responders				Histamine release				Numbers (*n*)			
High				>42.26%				17			
Ave				11.66–42.26%				63			
Low				<11.66%				14			
Ave + Low				<42.26%				77			
Total								94			

**Table 2:** Theoretical deduction of 15 possible combinatorial expression patterns of each individual sample derived by combining the observed expression levels of each of the 4 selected genes (AKAP12, ITM2C, IL13RA1, NELL2).

Up	Norm	Down	Combination	Category
4	0	0	4U0N0D	4-Up
3	1	0	3U1N0D	3-Up
2	2	0	2U2N0D	2-Up
1	3	0	1U3N0D	1-Up
0	4	0	0U4N0D	Others
3	0	1	3U0N1D	Others
2	1	1	2U1N1D	Others
1	2	1	1U2N1D	1-Up-1-Down
0	3	1	0U3N1D	Others
2	0	2	2U0N2D	Others
1	1	2	1U1N2D	Others
0	2	2	0U2N2D	Others
1	0	3	1U0N3D	Others
0	1	3	0U1N3D	Others
0	0	4	0U0N4D	Others

U = Up. N = Norm. D = Down.

**Table 3:** Combinatorial expression patterns of samples selected from the normal donor cohort (D#148, D#104, D#118) and allergic patient cohort (SH#1, SH#5, SH#7) were constructed by combining the observed expression levels (Up, Norm, Down) of each of the 4 selected genes (AKAP12, ITM2C, IL13RA1, NELL2) in tandem in each individual sample.

Sample ID	Histamine Release	Responder Group	NELL2	ITM2C	AKAP12	IL13RA1	Combination	Category
D#148	57.3%	High	Up	Up	Up	Up	4U0N0D	4-Up
D#014	20.4%	Ave	Norm	Down	Down	Norm	0U2N2D	Others
D#118	5.9%	Low	Norm	Norm	Norm	Norm	0U4U0D	Others
SH#1	11.5%	Ave	Norm	Norm	Norm	Norm	0U4N0D	Others
SH#5	36.3%	Ave	Norm	Down	Down	Norm	0U2N2D	Others
SH#7	44.2%	High	Up	Norm	Up	Up	3U1N0D	3-Up

## Data Availability

Restrictions apply to the availability of these data. Data were obtained from Apollonian Biosystems and are available from the corresponding author (S.-Y.T.) with permission from Apollonian Biosystems.

## Supplementary Materials

The following supporting information can be downloaded at: https://www.mdpi.com/article/10.3390/cells13151237/s1, Table S1: Direct correlation between microarray data and TaqMan q-PCR data on the association between differential gene expression levels and IgE-dependent activation responses among different human mast cell responders. Human mast cell cultures derived from normal donors were classified as High, Average or Low responders according to their responses to anti-IgE stimulation. RNA samples derived from unstimulated mast cell cultures from each of the 3 responder groups, respectively, were analyzed by microarray (*n* = 60 for mast cell cultures) and TaqMan q-PCR (*n* = 20 for each selected gene); Figure S1: Anti-IgE induced degranulation responses exhibited by human mast cell cultures that were selected from High (*n* = 22), Average (*n* =17), and Low (*n* = 21) responder groups for microarray gene expression analysis. Each dot of different colors shown in this figure represents a single normal individual donor
