# Supplementary material for: Combinatorial Genomic Biomarkers Associated with High Response in IgE-Dependent Degranulation in Human Mast Cells
Source: Cells. 2024 Jul 23;13(15):1237. doi: 10.3390/cells13151237 (PMC11311466; doi:10.3390/cells13151237)
Supplement: Supplementary file 1 [file cells-13-01237-s001.zip › cells-3090484-supplementary.pdf]

Supplementary Materials

Table S1. Direct correlation between microarray data and TaqMan q-PCR data on the association between differential gene expression levels and IgE-dependent activation responses among different human mast cell responders. Human mast cell cultures derived from normal donors were classified as High, Average or Low responders according to their responses to anti-IgE stimulation. RNA samples derived from unstimulated mast cell cultures from each of the 3 responder groups, respectively, were analyzed by microarray (n = 60 for mast cell cultures) and TaqMan q-PCR (n = 20 for each selected gene).

**Gene**	**Microarray Data**<br>**Changes in gene expression levels in High responders compared with those in Low responders**	**TaqMan q-PCR Data**<br>**Correlation curves between gene expression levels and IgE-dependent cell activation (Y = slope of the fitted trend line)**
**AKAP12**	High ↑ vs Low	3.647
**CALB2**	High ↑ vs Low	4.470
**DPP44**	High ↓ vs Low	-7.501
**FCGR1A**	High ↓ vs Low	-0.114
**FOXF1**	High ↑ vs Low	39.101
**GADD45B**	High ↓ vs Low	-24.602
**IL13RA1**	High ↑ vs Low	4.006
**IRF1**	High ↓ vs Low	-19.727
**ITM2C**	High ↑ vs Low	12.698
**NELL2**	High ↑ vs Low	4.523
**PTGDR**	High ↓ vs Low	-1.751
**SIGLEC8**	High ↓ vs Low	-12.877
**SOCS2**	High ↓ vs Low	-20.167
**SPTLC3**	High ↓ vs Low	-11.509
**TMEM255B**	High ↑ vs Low	14.381

Fig. S1

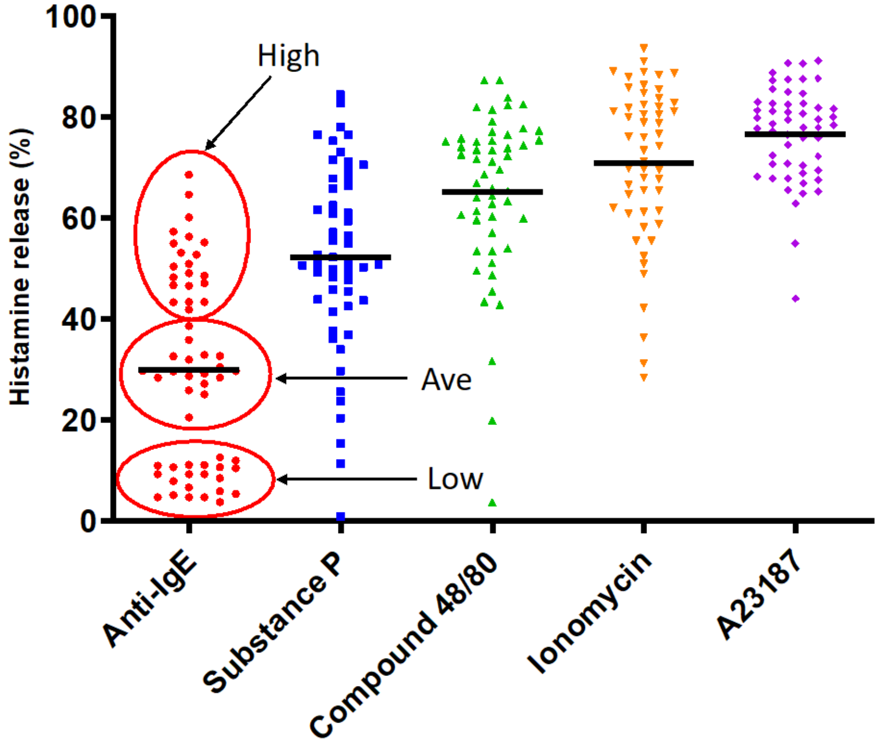

Figure S1. Anti-IgE induced degranulation responses exhibited by human mast cell cultures that were selected from High (*n* = 22), Average (*n* =17), and Low (*n* = 21) responder groups for microarray gene expression analysis. Each dot of different colors shown in this figure represents a single normal individual donor.
